# Homozygosity by descent mapping of blood pressure in the Old Order Amish: evidence for sex specific genetic architecture

**DOI:** 10.1186/1471-2156-8-66

**Published:** 2007-10-01

**Authors:** Patrick F McArdle, Harvey Dytch, Jeffery R O'Connell, Alan R Shuldiner, Braxton D Mitchell, Mark Abney

**Affiliations:** 1 Department of Medicine, University of Maryland School of Medicine, Baltimore, MD USA; 2 Department of Human Genetics, University of Chicago, Chicago, IL USA; 3 Geriatrics Research and Education Clinical Center, Veterans Administration Hospital Medical Center, Baltimore, MD USA

## Abstract

**Background:**

High blood pressure is a well established risk factor for morbidity and mortality acting through heart disease, stroke and cardiovascular disease. Genome wide scans have linked regions of nearly every human chromosome to blood pressure related traits. We have capitalized on beneficial qualities of the Old Order Amish of Lancaster, PA, a closed founder population with a relatively small number of founders, to perform a genome wide homozygosity by descent mapping scan. Each individual in the study has a non zero probability of consanguinity. Systolic and diastolic blood pressures are shown to have appreciable dominance variance components.

**Results:**

Areas of two chromosomes were identified as suggestive of linkage to SBP and 5 areas to DBP in either the overall or sex specific analyses. The strongest evidence for linkage in the overall sample was to Chromosome 18q12 (LOD = 2.6 DBP). Sex specific analyses identified a linkage on Chromosome 4p12-14 (LOD in men only = 3.4 SBP). At Chromosome 2q32-33, an area where we previously reported significant evidence for linkage to DBP using a conventional identity by descent approach, the LOD was 1.4; however an appreciable sex effect was observed with men accounting for most of the linkage (LOD in men only = 2.6).

**Conclusion:**

These results add evidence to a sex specific genetic architecture to blood pressure related traits, particularly in regions of linkage on chromosome 2, 4 and 18.

## Background

Hypertension is a common chronic condition in the United States and leads to severe morbidity and mortality through heart disease, stroke, congestive heart failure, end stage renal disease and peripheral vascular disease. Among these, heart disease and stroke are two of the leading cause of death in the United States [1,2]. Much effort and expense has been spent attempting to identify genes responsible for blood pressure variation and hypertension. At this point nearly all human chromosomes have been linked to hypertension related traits through linkage analysis [3-6]. It seems clear that a single gene is not responsible for hypertension and that etiology is a complex combination of both genetic and environmental risk factors [7,8]. Sex may be an important and easily identifiable "environmental" risk factor that may interact with putative genetic factors to increase risk [9].

To attempt to identify genetic causes to blood pressure variation and other complex diseases, we have studied a population of Old Order Amish (OOA) in Lancaster County, Pennsylvania. The OOA are a closed founder population with several beneficial qualities for the genetic study of complex diseases [10]. A relatively homogenous lifestyle tends to minimize variation of environmental risk factors associated with high blood pressure such as high fat diet and low physical activity levels. Furthermore, other risk factors such as smoking and alcohol consumption appear in very low levels if at all in the OOA community. Therefore we hypothesize that genetic determinants of complex diseases will be easier to identify in an OOA population where many environmental risk factors do not have wide variation.

The genetic ancestry of the OOA arises from a small number of founders, which reduces the genetic complexity and allows for special statistical analyses to be performed. The founders of the OOA population originated in Western Europe and immigrated to central Pennsylvania in the early 1700s [11]. A recent analysis of the OOA genealogy indicate that over 95% of the current gene pool is descendent from fewer than 100 individuals [12]. Marriage into the Amish community is rare and marriage within the Amish community tends to take place between members of geographically local church districts. Beginning with the founding of the Amish community in Pennsylvania, detailed pedigree records have been maintained allowing for the OOA population to be connected in a single 12–13 generation pedigree.

We previously capitalized on these advantages to map a quantitative trait locus influencing blood pressure to chromosome 2q31-34 [13]. Our mapping approach in that report utilized only a portion of the available 12–13 generation pedigree structure in testing whether the probability of sharing alleles identical by descent (IBD) across relative pairs was correlated with their degree of phenotypic similarity. In this report, we take more full advantage of the relatively small number of OOA founders and the detailed pedigree information in order to assess consanguineous relationships and carry out Homozygosity By Descent (HBD) mapping. In contrast to the more common IBD linkage analysis, where sharing between individuals is used, HBD mapping looks strictly at sharing within an individual and correlates phenotypic values with the probability of an individual having two copies of an ancestral allele at a given locus. Thus HBD mapping is specifically designed to detect recessive acting alleles originating in a founder that possibly lead to an altered phenotypic expression. It has recently been estimated that areas of HBD are more common than one would expect in affected individuals from consanguineous relationships, which would only act to increase the power of our approach [14]. We present here the results of HBD mapping of the continuous traits systolic and diastolic blood pressure (SBP and DBP, respectively) among a sample of OOA individuals. Results from sex specific linkage analysis are also presented. We additionally compare the HBD mapping results with those obtained from the previous IBD linkage analysis of these traits in this same population.

## Results

The study sample consisted of 616 individuals, 31 of whom were prescribed anti-hypertensive medication at the time of the study. The age of individuals ranged from 18 to 93 with a mean of 47.4 years. The individuals in the study had a mean BMI of 27.3 (standard deviation = 4.9). There were 336 (54.6%) women included.

Previous estimates of the heritability of SBP and DBP (0.23 and 0.29 respectively) given for this sample of OOA were of the narrow sense, or the proportion of phenotypic variation due to the additive effect of genes [13]. Residual heritability estimates after accounting for age, age^2 ^and sex with both additive and dominance variance proportions are given in Table 1 for the four blood pressure traits. Included in this table are also the heritability estimates of the naive blood pressure traits if anti-hypertensive medication use was ignored.

**Table 1:** Proportion of trait variance (standard error) attributable to the additive and dominance effect of genes and the residual variance attributed to the environment among the OOA, Lancaster County PA

			Proportion of Variance		
			
Trait	Number	Mean (SD) mmHg	Additive	Dominance	Environmental
With those on medication removed					
SBP	585	121.3 (15.9)	0.14 (0.12)	0.32 (0.28)	0.54 (0.21)
DBP	585	78.3 (9.2)	0.17 (0.12)	0.25 (0.25)	0.59 (0.18)
Adding a constant to those on medication					
SBP	616	123.6 (19.1)	0.04 (0.13)	0.96 (0.13)	0.00
DBP	616	79.0 (10.1)	0.16 (0.11)	0.46 (0.27)	0.38 (0.21)
Ignoring medication use					
SBP	616	123.1 (18.1)	0.08 (0.12)	0.76 (0.33)	0.17 (0.25)
DBP	616	78.8 (9.8)	0.17 (0.11)	0.37 (0.26)	0.46 (0.20)

Our HBD mapping identified 2 chromosomal regions associated with SBP with a LOD score of 2.0 or greater in either the total combined sample or men and women separately. Similarly, 5 regions were linked to DBP. These results are given in Table 2.

**Table 2:** Genomic areas associated with blood pressure related traits with a LOD score of 1.5 or greater by HBD mapping among the OOA, Lancaster County PA

		Overall		Men		Women	
		
Chromosome	Peak Position (cM)	Removed	Adjusted	Removed	Adjusted	Removed	Adjusted
Systolic Blood Pressure							
3	167	1.6		2.0	1.8		
4	64		2.0	3.0	3.4		
							
Diastolic Blood Pressure							
2	220			2.5	2.7		
3	128					2.3	
16	102					2.3	1.6
18	43			2.0	1.8		
18	53–55	2.6	2.2	1.8	1.6		

Removed = those on medication removed from analysis.

Adjusted = constant added to measured blood pressure of those on medication.

**Figure 1:**
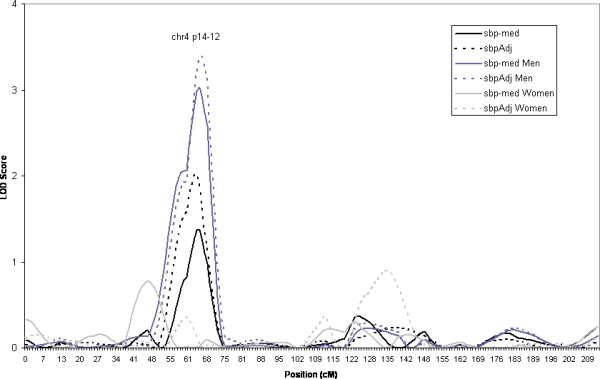
Estimated LOD score obtained from HBD mapping of SBP on Chromosome 4. Linkage results are given for the overall sample and men and women separately. (sbp-med: systolic blood pressure with those on medication removed, sbpAdj = systolic blood pressure adjusted for medication as described in text).

Our highest LOD score was found on chromosome 4p14-12 to SBP (LOD = 3.4). Modest evidence for linkage also exists to DBP in this area among men (LOD = 1.7). The linkage in this area is present in men only, see Figure 1. There is little to no evidence for linkage to either SBP or DBP in this region among women.

**Figure 2:**
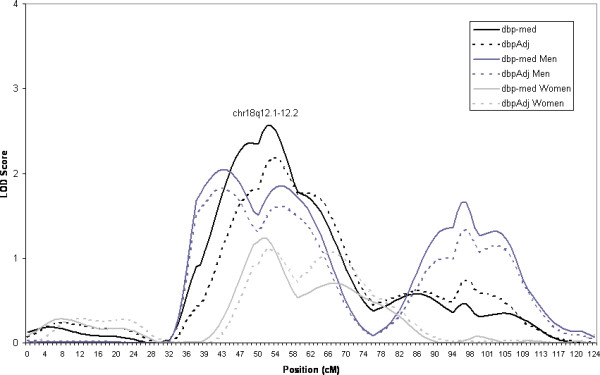
Estimated LOD score obtained from HBD mapping of DBP on Chromosome 18. Linkage results are given for the overall sample and men and women separately. (dbp-med: diastolic blood pressure with those on medication removed, dbpAdj = diastolic blood pressure adjusted for medication as described in text).

Our highest LOD score for the combined sample (i.e. both sexes) was on chromosome 18, figure 2. The linkage is to DBP (LOD = 2.6). There is evidence for linkage in both sexes, although to different regions on chromosome 18. The linkage heterogeneity between the sexes may be responsible for the wide peak seen in the overall sample.

**Figure 3:**
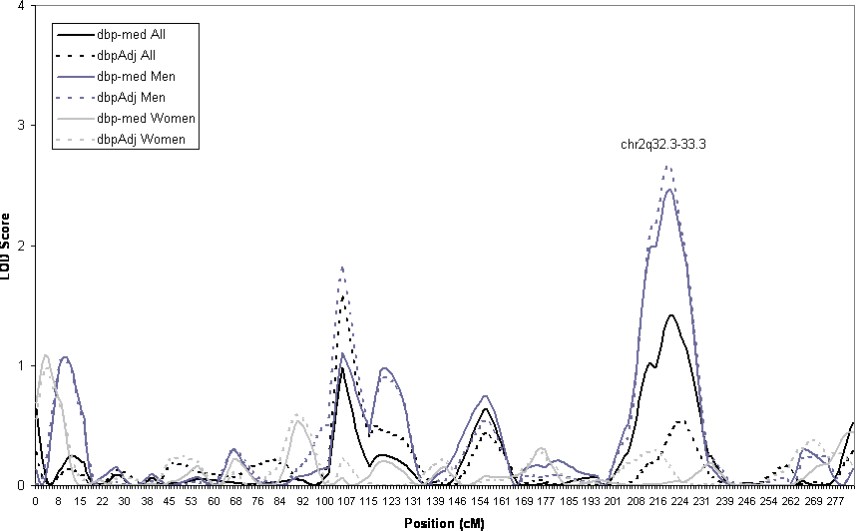
Estimated LOD score obtained from HBD mapping of DBP on Chromosome 2. Linkage results are given for the overall sample and men and women separately. (dbp-med: diastolic blood pressure with those on medication removed, dbpAdj = diastolic blood pressure adjusted for medication as described in text).

Our previous linkage analysis in this sample identified a region on chromosome 2q 31-34 linked to DBP [13]. The current HBD mapping analysis identified the same region on chromosome 2 with a LOD score = 1.4 in the overall sample with the trait DBP with those on medication removed, the same trait as initially identified, see Figure 3. This locus also seems to have significant sex heterogeneity in the linkage signal, with the linkage in the region being almost entirely attributable to men. The LOD score = 2.5 when only men are considered in the analysis.

## Discussion

Isolated populations have been proposed to be particularly valuable to the mapping of complex traits [15]. We have used a HBD mapping technique here that is particularly suited for such an isolated population. Our previous results based upon identity by descent linkage analysis allow the opportunity to compare directly the two techniques.

Our approach to HBD mapping has been used to identify genetically linked loci to fasting serum insulin and triglyceride levels in a population of Hutterites, a closed founder population in South Dakota [16,17]. This study is the first use of these methods in a population outside of the Hutterites. The OOA of Lancaster Pennsylvania and the Schmiedeleut Hutterites of South Dakota have similar pedigree structures and levels of consanguinity. Estimates of inbreeding and kinship coefficients found here in the OOA are similar to those identified in the Hutterites [18]. The success of these mapping efforts may indicate that methods developed for either population may be applicable and are computationally manageable in other comparable closed founder populations.

We further investigated the evidence for linkage by plotting the multi-locus probability of HBD against residual trait values (data not shown). Since homozygosity (by state) is observable, we were able to assess the genotypes of the individuals who had high probability of HBD at a locus and extreme phenotypic values. We were unable to identify strong evidence for a founder effect in the data.

Our estimates of broad heritability of SBP warrant further investigation. They indicate that the method used to account for hypertension medication may have large effects when estimating variance components of blood pressure variation. Adding a constant to the measured values of medicated individuals is the preferred method of some [19,20]. Our results indicate that after accounting for age and sex, the residual variance of SBP adjusting for medication in this way is almost entirely under genetic control, which is unlikely since measurement error alone should account for at least some environmental component. It should be noted that the standard errors of these estimates are rather large. Analysis of dominance trait variance in the Hutterites found a significant dominance component to SBP as well and selected a best fitting model where the additive component was small. In addition, other traits, including LDL and Serotonin levels, had little to no environmental component in the Hutterites [18].

Estimates of consanguinity and HBD may be underestimated in this analysis. The OOA pedigree available is 12–13 generations deep and dates back only to the Amish immigration from Europe to central Pennsylvania. It assumes all founders, defined as those individuals whose parents are not represented in the pedigree, are unrelated. This assumption is conservative, as some of the Amish founders who emigrated together from Europe may in fact have been related.

The original ascertainment scheme of our cohort was based upon the identification of diabetic probands of whom family members were recruited into the study. Therefore it is possible that our cohort is enriched for diabetes relevant alleles. Our linkage results presented here could correspondingly be interpreted as identifying genes that are linked to blood pressure particularly in the presence of diabetes susceptibility variants. Interestingly, the p arm of chromosome 4 has previously been linked to SBP, as well as to plasma free fatty acid levels, in a sample of Dutch dyslipidemic families [21], which provides evidence that the association between genes in the region and blood pressure regulation maybe act through insulin resistance pathways. For this reason, we choose not to adjust for diabetic status in our analysis, since we are interested in finding genes that act on blood pressure, even those who act primarily via a diabetes related pathway.

We have performed genome wide HBD analysis for four blood pressure traits in our sample and in strata defined by sex. These four traits are correlated and the sex strata overlap with the overall sample analysis. Correspondingly, we present here nominal LOD scores and did not correct for multiple comparisons between related traits or overlapping samples.

It has been suggested that many complex quantitative traits in humans may exhibit a sex specific genetic architecture [9]. Our results are consistent with this hypothesis and suggest that blood pressure related traits may have a sex specific genetic architecture. The present HBD investigation identified a similar region on chromosome 2 as the previous IBD linkage analysis [13] and sex specific analysis indicates that this linkage may be due to recessive acting alleles in men. Sex specific differences were also seen in two other regions with evidence for linkage, on chromosomes 4 and 18. The sex specific results on chromosome 18 provide enlightening results. The linkage peak in the overall sample was our most significant result but was extremely wide, a one LOD interval covering over 22 cM. Sex specific results identified a narrow peak in the region among women and two flanking peaks among men. The male specific linkage peak q-ter of the overall peak, overlaps with a previously identify region linked to essential hypertension in Icelandic families [22]. The sex specific refinement of the linkage peak may aid in the identification of responsible genes in the region.

We have identified a similar region on chromosome 2 as the previously reported identity by descent linkage in this population. We hesitate to term this second finding as a "validation" or a "replication" of the initial result because these are indeed two different analysis asking slightly different questions. In a traditional identity by descent linkage, regions of the chromosome are identified that related individuals with similar trait values share identical by descent more often than expected by chance. The fundamental analysis in IBD linkage is between individuals. In the HBD linkage, we are looking within an individual to find areas of the genome where an individual's two chromosome are shared identical by descent (thus homozygosity by descent) and then ask whether this region is linked with a trait of interest. Therefore by definition the HBD analysis is designed to map recessive acting genetic effects. So how should we interpret an area of the genome that supplies evidence of linkage by both methods? One possibility is there are multiple genes in the region or perhaps multiple mutations within a single gene that act in different manners and that the two analyses are identifying two different genetic effects in the same region. Alternatively, the two methods may be identifying the same genetic effect, which is strong enough to be detected by the two different approaches.

The converse of this question is how to interpret the results that identify a region of the genome by one analysis but not the other. We found this scenario on chromosome 18q. The HBD analysis presented here returned a LOD of greater than 2 to DBP. Our initial linkage results found no such evidence on chromosome 18. It is possible that the genetic effect in this region acts in a purely recessive manner but is not strong enough to be identified by identity by descent linkage. Alternatively, the possibility must also be considered that our HBD linkage in this region is a false positive result or that the identity by descent linkage is a false negative, each of which would lead to drastically different conclusions.

## Conclusion

How to interpret results from linkage analysis will eventually come to rest upon issues of a practical nature. Ideally resources would be available to track down all suggested areas of linkage with further analysis (fine mapping, candidate gene studies, linkage disequilibrium mapping, etc.). In the case of hypertension and blood pressure related traits, this would mean investigating nearly every human chromosome [6]. Since resources are limited, regions should be identified that hold greatest evidence across populations and with a variety of analytic techniques. The q arm of chromosome 2 is one such region identified, particularly to DBP in Caucasian populations [3]. We present here further evidence for linkage to chromosome 2q in a population of OOA utilizing a HBD mapping approach.

## Methods

The Amish Family Diabetes Study began in 1995 with the goal of identifying susceptibility genes for type 2 diabetes and related traits [23]. The protocol was approved by the Institutional Review Board of the University of Maryland. Informed consent was obtained in writing prior to participation. Probands were individuals identified with type 2 diabetes and family members over the age of 18 were recruited into the study. The study protocol was carried out either in the individual's home or in the Amish Diabetes Research Clinic in Strasburg, Pennsylvania. During the study visit multiple phenotypes were ascertained, including blood pressure measurements. Systolic (first phase) and diastolic (fifth phase) blood pressure (SBP and DBP respectively) were obtained in duplicate by the use of a standard sphygmomanometer with the subject sitting for at least 5 minutes and was recorder to the nearest mmHg.

The use of anti-hypertensive medication is rare in the OOA community, but it does exist. In our study sample, 31 individuals out of the 616 with blood pressure measurements self-reported anti-hypertension medication use, only 5% of the sample. We accounted for anti-hypertensive medication use in these analyses using two different approaches. First, we removed those on medication from the analysis (annotated bp-med). This method of accounting for medication was used in the previous study of blood pressure in this population [13] and was chosen because anecdotal evidence suggest that compliance to prescription guidelines are not rigid in the OOA community and members tend not to control their blood pressure tightly with medication. Therefore true underlying blood pressure levels would be difficult to predict. The second method used to account for medication was to add a constant to the blood pressure measurements of those prescribed medication (annotated bpAdj). Based on the effects of anti-hypertensive medication from a double blind randomized controlled trial [24], Cui et al. (2003) recommend adding a constant of 10 mmHg to SBP and 5 mmHg to DBP to the observed blood pressures of those on medication [19]. This method of adjustment for medication has been demonstrated to perform well in a range of realistic clinical scenarios [20]. Trait residuals were checked and they were approximately normally distributed and no outliers were identified.

Participants were genotyped using the ABI Prism Linkage Mapping Set (Perkin-Elmer), a screen set of 357 high polymorphic short tandem repeat markers spaced across the genome at an average distance of 10 cM. The largest gap between markers is 25.4 cM on chromosome 7. The markers used here are identical to those used in the previous linkage study of blood pressure in this population [13].

### Statistical Methods

Variance components were used to estimate heritabilities of the traits. Jacquard's condensed identity coefficients were computed using an algorithm accounting for the large inbred population [25,26]. A matrix of Jacquard's seventh identity coefficient (Δ7) was specified to estimate the proportion of phenotypic variation attributable to the dominance effect. The i^th^, j^th ^element in the matrix gives the probability that individuals i and j share two alleles IBD and neither of the individuals is autozygous. Polygenic additive and dominance variances estimates were made using the SOLAR software package (Southwest Foundation for Biomedical Research, San Antonio TX).

Genome wide scans were performed using HBD mapping methods and software developed by Abney et. al. and complete statistical methods are described elsewhere [17]. In brief, these methods rely on a non-zero probability of an individual's parents sharing at least one common ancestor. The conditional probability of being HBD is based upon the complete pedigree information and an individual's multi-locus genotype data and is computed via a hidden Markov model method. The HBD probability is included in a linear model as a fixed effect. The linear model also contains additive and dominance polygenic variance components. Thus the final model was:

Y = Xβ + γh + g + e

where Y is a vector of individual phenotype values. X is a design matrix accommodating fixed environmental covariates and β is a vector of corresponding effect parameters. The h vector contains the multi-point estimates of HBD at the locus and the γ term is the effect of interest. The g term is the polygenic component that is distributed multivariate normally with a mean of zero and a covariance equal to two times the kinship matrix (Φ) times the expected variance due to the additive effect of genes plus Δ7 matrix times the expected dominance variance.

g ~MVN(0, 2Φσ_a_^2 ^+ Δ7σ_d_^2^)

The e term is a normally distributed error component with mean zero and variance equal to σ_e_^2^.

The significance of the HBD effect (H_o_: γ = 0) was assessed at 1 cM intervals across the genome. Because the method computes p-values directly, to obtain equivalent LOD scores the nominal p-values were converted to a corresponding chi-square statistic with one degree of freedom and divided by twice the natural logarithm of 10. Each model included covariate terms for sex, age and age^2^. The sex specific results presented are obtained by including individuals of one sex only in the analysis.

Sex and age were the only covariates considered for this analysis for primarily two reasons; firstly, to avoid making any a priori assumptions regarding the causal mechanisms between the genes and blood pressure levels. Adjusting for covariates that are associated with both the exposure (major genes) and the outcome (blood pressure) only makes sense if the covariate is confounding the association. It is just as likely that any potential covariate could be on the causal pathway between the major gene and blood pressure and adjusting for it in the analysis will increase type II error rate (i.e. produce a false negative). Since sex can be safely assumed to not lie on any casual pathway (e.g. it would be difficult to envision a scenario were a gene "causes" sex), stratifying the results by sex could add additional insight into major genes effect on blood pressure. Finally, and of secondary importance, one aim of this paper was to compare the results from the HBD mapping exercise directly with the IBD linkage results previously reported in this sample and thus the same covariates were used so that a direct comparison would be valid.

To construct the required pedigree structures for the HBD calculations, we started with the 616 individuals with measured blood pressure values participating in the AFDS, and then added all possible ancestors present in the Anabaptist Genealogy Database (AGDB) version 3.0 [27]. This process resulted in a single pedigree comprising 6308 individuals. Based on this pedigree the 616 individuals have a mean (standard deviation) coefficient of consanguinity of 0.033 (0.012), and pairwise Δ7 ranging from 2.11 × 10^-5 ^to 0.267 with mean 0.0041. For outbred populations Δ7 is 0.25 for siblings.

## Authors' contributions

PFM participated in the design of the analysis, performed the statistical analysis and drafted the manuscript. HD contributed to writing the analysis software and in the statistical analysis. JRO assisted in the interpretation of the results and the statistical analysis. ARS designed the study, oversaw the recruitment of subjects and conduct much of the primary data gathering. BDM participated in the design of the study, the interpretation of the results and drafting of the manuscript. MA developed the HBD methods, assisted in the statistical analysis and implementation of the analytic methods. All authors read and approved the final manuscript.

## References

[B1] Stamler J (1991). Blood pressure and high blood pressure. Aspects of risk. Hypertension.

[B2] WISQARS WISQARS Leading Causes of Death Reports, 1999 - 2002. http://webapp.cdc.gov/sasweb/ncipc/leadcaus10.html.

[B3] Koivukoski L, Fisher SA, Kanninen T, Lewis CM, von Wowern F, Hunt S, Kardia SL, Levy D, Perola M, Rankinen T, Rao DC, Rice T, Thiel BA, Melander O (2004). Meta-analysis of genome-wide scans for hypertension and blood pressure in Caucasians shows evidence of susceptibility regions on chromosomes 2 and 3. Hum Mol Genet.

[B4] Samani NJ (2003). Genome scans for hypertension and blood pressure regulation. Am J Hypertens.

[B5] Garcia EA, Newhouse S, Caulfield MJ, Munroe PB (2003). Genes and hypertension. Curr Pharm Des.

[B6] Mein CA, Caulfield MJ, Dobson RJ, Munroe PB (2004). Genetics of essential hypertension. Hum Mol Genet.

[B7] Lifton RP (1996). Molecular genetics of human blood pressure variation. Science.

[B8] Hamet P, Pausova Z, Adarichev V, Adaricheva K, Tremblay J (1998). Hypertension: genes and environment. J Hypertens.

[B9] Weiss LA, Pan L, Abney M, Ober C (2006). The sex-specific genetic architecture of quantitative traits in humans. Nat Genet.

[B10] McKusick VA (1978). Medical genetic studies of the Amish : selected papers.

[B11] Cross HE (1976). Population studies and the Old Order Amish. Nature.

[B12] Pollin T (2004). The Old Order Amish of Lancaster County : elucidating the male founder structure and mapping metabolic syndrome quantitative.

[B13] Hsueh WC, Mitchell BD, Schneider JL, Wagner MJ, Bell CJ, Nanthakumar E, Shuldiner AR (2000). QTL influencing blood pressure maps to the region of PPH1 on chromosome 2q31-34 in Old Order Amish. Circulation.

[B14] Woods CG, Cox J, Springell K, Hampshire DJ, Mohamed MD, McKibbin M, Stern R, Raymond FL, Sandford R, Malik Sharif S, Karbani G, Ahmed M, Bond J, Clayton D, Inglehearn CF (2006). Quantification of homozygosity in consanguineous individuals with autosomal recessive disease. Am J Hum Genet.

[B15] Shifman S, Darvasi A (2001). The value of isolated populations. Nat Genet.

[B16] Newman DL, Abney M, Dytch H, Parry R, McPeek MS, Ober C (2003). Major loci influencing serum triglyceride levels on 2q14 and 9p21 localized by homozygosity-by-descent mapping in a large Hutterite pedigree. Hum Mol Genet.

[B17] Abney M, Ober C, McPeek MS (2002). Quantitative-trait homozygosity and association mapping and empirical genomewide significance in large, complex pedigrees: fasting serum-insulin level in the Hutterites. Am J Hum Genet.

[B18] Abney M, McPeek MS, Ober C (2001). Broad and narrow heritabilities of quantitative traits in a founder population. Am J Hum Genet.

[B19] Cui JS, Hopper JL, Harrap SB (2003). Antihypertensive treatments obscure familial contributions to blood pressure variation. Hypertension.

[B20] Tobin MD, Sheehan NA, Scurrah KJ, Burton PR (2005). Adjusting for treatment effects in studies of quantitative traits: antihypertensive therapy and systolic blood pressure. Stat Med.

[B21] Allayee H, de Bruin TW, Michelle Dominguez K, Cheng LS, Ipp E, Cantor RM, Krass KL, Keulen ET, Aouizerat BE, Lusis AJ, Rotter JI (2001). Genome scan for blood pressure in Dutch dyslipidemic families reveals linkage to a locus on chromosome 4p. Hypertension.

[B22] Kristjansson K, Manolescu A, Kristinsson A, Hardarson T, Knudsen H, Ingason S, Thorleifsson G, Frigge ML, Kong A, Gulcher JR, Stefansson K (2002). Linkage of essential hypertension to chromosome 18q. Hypertension.

[B23] Hsueh WC, Mitchell BD, Aburomia R, Pollin T, Sakul H, Gelder Ehm M, Michelsen BK, Wagner MJ, St Jean PL, Knowler WC, Burns DK, Bell CJ, Shuldiner AR (2000). Diabetes in the Old Order Amish: characterization and heritability analysis of the Amish Family Diabetes Study. Diabetes Care.

[B24] Neaton JD, Grimm RH, Prineas RJ, Stamler J, Grandits GA, Elmer PJ, Cutler JA, Flack JM, Schoenberger JA, McDonald R (1993). Treatment of Mild Hypertension Study. Final results. Treatment of Mild Hypertension Study Research Group. Jama.

[B25] Jacquard A (1974). The genetic structure of populations.

[B26] Abney M, McPeek MS, Ober C (2000). Estimation of variance components of quantitative traits in inbred populations. Am J Hum Genet.

[B27] Agarwala R, Biesecker LG, Schaffer AA (2003). Anabaptist genealogy database. Am J Med Genet C Semin Med Genet.

